# Machine Learning-Based Pipette Positional Correction for Automatic Patch Clamp *In Vitro*

**DOI:** 10.1523/ENEURO.0051-21.2021

**Published:** 2021-07-23

**Authors:** Mercedes M. Gonzalez, Colby F. Lewallen, Mighten C. Yip, Craig R. Forest

**Affiliations:** Georgia Institute of Technology, George W. Woodruff School of Mechanical Engineering, Atlanta, GA 30332

**Keywords:** automated, CNN, deep learning, electrophysiology, machine learning, patch clamp

## Abstract

Patch clamp electrophysiology is a common technique used in neuroscience to understand individual neuron behavior, allowing one to record current and voltage changes with superior spatiotemporal resolution compared with most electrophysiology methods. While patch clamp experiments produce high fidelity electrophysiology data, the technique is onerous and labor intensive. Despite the emergence of patch clamp systems that automate key stages in the typical patch clamp procedure, full automation remains elusive. Patch clamp pipettes can miss the target cell during automated experiments because of positioning errors in the robotic manipulators, which can easily exceed the diameter of a neuron. Further, when patching in acute brain slices, the inherent light scattering from non-uniform brain tissue can complicate pipette tip identification. We present a convolutional neural network (CNN), based on ResNet101, to identify and correct pipette positioning errors before each patch clamp attempt, thereby preventing the deleterious effects of and accumulation of positioning errors. This deep-learning-based pipette detection method enabled superior localization of the pipette within 0.62 ± 0.58 μm, resulting in improved cell detection success rate and whole-cell patch clamp success rates by 71% and 59%, respectively, compared with the state-of-the-art cross-correlation method. Furthermore, this technique reduced the average time for pipette correction by 81%. This technique enables real-time correction of pipette position during patch clamp experiments with similar accuracy and quality of recording to manual patch clamp, making notable progress toward full human-out-of-the-loop automation for patch clamp electrophysiology.

## Visual Abstract

**Figure:**
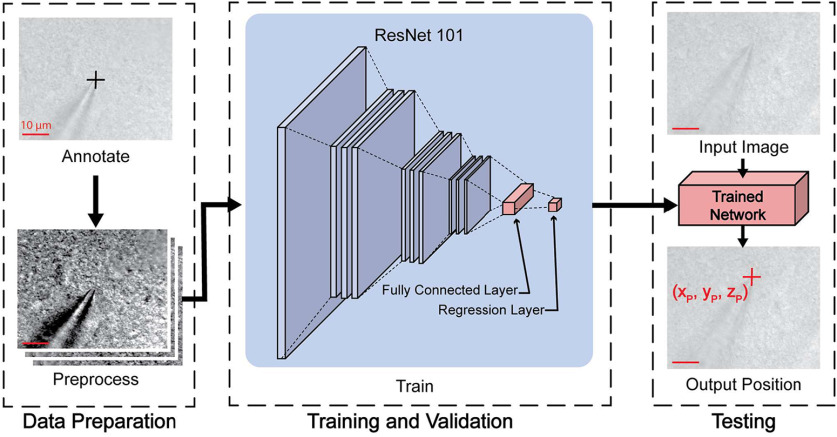

## Significance Statement

The patch clamp technique, while difficult and time intensive, remains necessary for fully elucidating individual neuron behavior. This deep-learning based method for pipette correction will improve the yield and speed of automated patch clamp experiments, enabling higher throughput and real-time pipette correction during fully automated patch clamp experiments.

## Introduction

Characterizing neuronal function on a single cell level is crucial to unraveling the biological mechanisms underlying brain activity. One of the most important techniques used in neuroscience to understand individual neuron behavior is patch clamp electrophysiology. This Nobel prize-winning technique allows one to record subthreshold current and voltage changes, enabling scientists to better understand neuronal communication. While optical methods offer a promising non-invasive method to study single neurons (Hochbaum et al., 2014; Kiskinis et al., 2018; Adam et al., 2019; Fan et al., 2020), their reliance on relative measurements rather than absolute voltage or current and suboptimal spatiotemporal resolution still require patch clamp to validate recordings of individual cellular behavior.

Typically, an *in vitro* patch clamp experiment is performed as follows: one views a brain slice under a microscope, manually maneuvers and delicately places a 1- to 2-μm tip of a glass pipette into contact with a 10-μm diameter cell membrane, creates a high-resistance seal between the pipette and cell membrane, and breaks into the cell to create a whole-cell configuration. This technique is immensely time intensive even for a skilled expert under optimal conditions. To improve the throughput and yield of these essential yet challenging experiments, several groups have invented automated patch clamp rigs for both *in vitro* (Wu et al., 2016; Kolb et al., 2019; Lewallen et al., 2019) and *in vivo* (Kodandaramaiah et al., 2012; Kolb et al., 2013; Annecchino et al., 2017; Stoy et al., 2017; Suk et al., 2017; Holst et al., 2019) electrophysiology, including a handful of techniques developed specifically for automated pipette localization (Long et al., 2015; Koos et al., 2017, 2021) and cell tracking (Lee et al., 2018).

One of the most challenging steps to automate in these rigs is the accurate and repeatable placement of the pipette tip close to the membrane of a cell (Long et al., 2015). Conventionally, patch pipettes are controlled by micromanipulators that have random and systematic errors on the order of 10 μm (Kolb et al., 2019) when repeatedly moving to and from the same location. A major drawback for previous pipette tip localization techniques (Long et al., 2015; Koos et al., 2017, 2021) is that the accuracy is significantly reduced when real-world background lighting variation and noise is introduced. Light scattering from the brain tissue induces significant noise in the image and renders these methods practically useless since they rely on a clear image of the pipette in acute slice experiments, despite their success in cultured cell experiments. To overcome this obstacle, we implemented a convolutional neural network (CNN), ResNet101, to automatically identify and correct the pipette tip localization error for automated *in vitro* patch clamp experiments. This method will not only improve the precise placement of the pipette near the cell membrane, but also reduce the time required to localize the pipette tip over a cell and therefore improve the overall throughput and efficiency of the automated patch clamp process.

## Materials and Methods

### Coordinate system and definition of errors

To accurately identify the pipette location for patch clamp experiments, we defined a coordinate system relative to the objective location so that the center of the field of view, with the pipette tip in focus, was considered the origin. Hereafter, the view of the brain slice under the microscope will be referred to as the *xy*-plane. The *z* direction is defined by the vertical distance perpendicular to that plane, with z = 0 at the location where the pipette was perfectly in focus.

**Figure 1.**
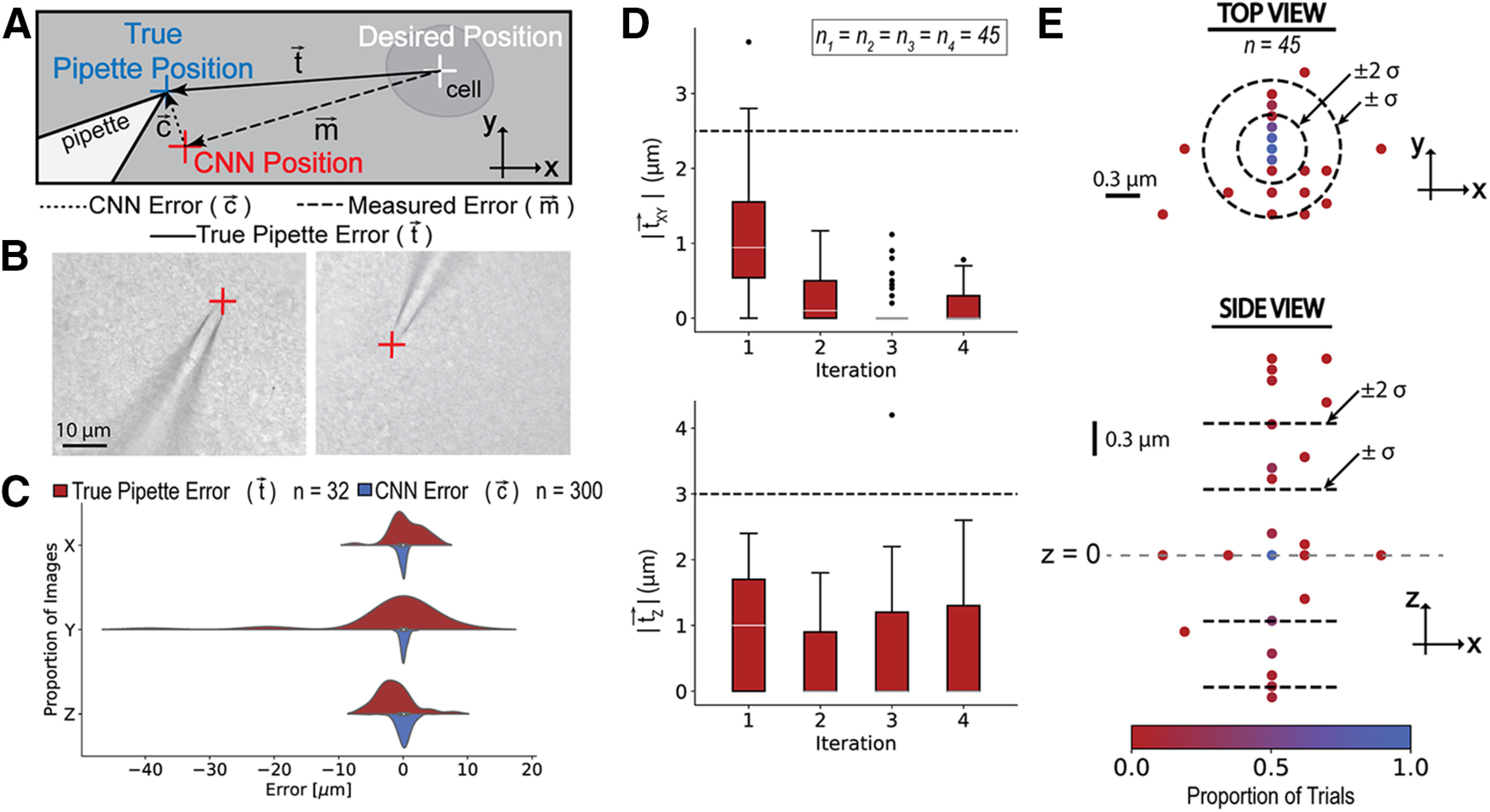
***A***, Schematic of the error nomenclature used in this work. ***B***, Example images of the CNN identifying the pipette tip over a brain slice. ***C***, Error distribution of neural network testing dataset *n* = 300 images (red) compared with the true pipette error after moving to the cleaning bath for *n* = 32 images (blue). ***D***, Convergence of true pipette error magnitude using the CNN as the measurement feedback in the (top) xy-plane and (bottom) *z* direction. The black dotted lines indicate appropriate error ranges for patch clamp experiments, at 2.5 mm in the xy-plane (half the diameter of a typical cell) and 3 mm in the z direction. The box width indicates the first and third quartiles, the white line indicates the median, and the whiskers of the box plot indicate the most extreme, non-outlier data points. ***E***, Spatial representation of pipette tip locations after the second iteration of using the CNN for correction in the (top) *xy*-plane and (bottom) *z* direction. Black dotted lines indicate the range of one and 2 SDs.

There were three types of positioning errors addressed, as shown in Figure 1*A*. When moving the pipette, the pipette is commanded to move to the desired position (white), typically coincident with the center of a cell (*x*,*y*)*_cell_* in automated patch clamp experiments. Because of random and systematic errors in the three-axis manipulators, the true pipette position (blue) is not equal to the desired position, resulting in the true pipette error ($\vec{t_{x}},\;\vec{t_{y}},\;\vec{t_{z}}$). We can estimate the true position of the pipette with the CNN, resulting in the computed CNN position (red). This CNN position has CNN error vector ($\vec{c_{x}},\;\vec{c_{y}},\;\vec{c_{z}}$), defined by the difference between the true pipette position and the CNN position. Since we cannot determine the true pipette position during an automated patch clamp experiment, we must use the CNN position as a feedback signal. Thus, we use the difference between the desired position and the CNN position, called the measured error ($\vec{m_{x}},\;\vec{m_{y}},\;\vec{m_{z}}$), to correct the pipette’s position.

### Image collection

The image datasets used for training, validation, and testing in this work consisted of 1024 × 1280 eight-bit raw images. We used a standard electrophysiology setup (SliceScope Pro 3000, Scientifica Ltd) with PatchStar micromanipulators at a 24° approach angle. We used a 40× objective (LUMPFLFL40XW/IR, NA 0.8, Olympus) and Rolera Bolt camera (QImaging), illuminated under DIC with an infrared light-emitting diode (Scientfica). The resulting field of view was 116 × 92 μm. All animal procedures were done in accordance with the National Institutes of Health *Guide for the Care and Use of Laboratory Animals* and the Georgia Institute of Technology animal care committee’s regulations.

### Neural network training, validation, and testing data

To construct a representative dataset of pipette images, images of 3–5 MΩ (1- to 2-μm diameter tip) pipettes were collected over a plain background as well as with a brain slice. The motivation for this is to ensure that the network would be robust enough to identify pipettes in both scenarios, if necessary. The ground truth annotation process began by sending a pipette to a computer-generated randomized location in the *xy*-plane (±27 μm). The user manually annotated the location of the pipette tip, in pixels, and confirmed the pipette was in focus so that the pipette could be imaged at fixed intervals along the *z*-axis at this position in the *xy*-plane. The pipette would then automatically move down (only in the *z* direction) with a constant step size to a random lower limit distance of up to 100 μm, collecting images at each step and recording the manually annotated *xy* location and prescribed *z* location (based on step size) as an (*x*,*y*,*z*) coordinate in pixels. The step sizes were constant for each *xy* location, but randomized (within 5–20 μm) in between. Once at the lower limit distance, the pipette would return to the in-focus position (Z = 0) at the same *xy* location. To ensure that the pipette tip location was accurate, the user would again manually annotate the tip, saving the (*x*,*y*) coordinate in pixels, while in focus. The pipette would then step in the positive *z* direction, collecting images and recording coordinates at each step until reaching an upper limit distance. A total of 6678 raw annotated images were captured for training, validation, and testing datasets. All training and testing data will be available at https://autopatcher.org/.

### Image preprocessing

All images used for training and validation were preprocessed using contrast stretching (Gonzalez and Woods, 2018) to improve the ability to identify the pipette tip. To accomplish this, we calculated the average $\left(\bar{x}\right)$ and SDs (*σ*) of the pixel intensities of each image and mapped the original pixel values to the range defined by $\left(\bar{x}\pm 2\sigma \right)$ for each image individually. This mapping improved the contrast by reducing the range of pixel intensities, thereby making a smaller range of pixel intensities more. Any pixel intensity that was outside the range of [0,1] after mapping was set to 0 or 1, respectively. The images were then cropped to a square from the center and downsized to 224 × 224 for use with the CNN. These images were then transformed to artificially increase the training dataset, making the network more robust to different orientations of the pipette. The images, and their corresponding pipette tip location annotations, were flipped horizontally, vertically, and both horizontally and vertically. These three augmentations resulted in a total training dataset of 24,747 images and a validation dataset of 765 images. The test images underwent the same preprocessing, but no augmentations, resulting in a total of 300 test images. All preprocessing and network training was done using MATLAB 2020a and all patch clamp experiments were done with MATLAB and LabVIEW programs.

### CNN training

The pretrained network model, ResNet101, was used as the basis for this work. ResNet101 is a CNN, 101 layers deep, that is trained for classification problems. The residual network family is known for performing well in classification challenges because the depth of these CNNs lead to superior performance (Zhang et al., 2018). Here, we wanted to predict the (*x*,*y*,*z*) location of the pipette tip based on an image. To accomplish this, we replaced the final three layers of the ResNet101 architecture with a fully connected layer and a regression layer (Lathuiliere et al., 2020). This allowed us to define the output as a continuous 3 × 1 vector, corresponding to the (*x*,*y*,*z*) location of the pipette tip.

The training options are summarized in Table 1. Of the optimizers available in the MATLAB Deep Learning Toolbox, the *rmsprop* (root mean square propagation) optimizer, or loss function, has reported the greatest accuracy (Vani and Rao, 2019). The mini batch size should be a power of 2 and maximized for accuracy (Goodfellow et al., 2016). While computer RAM availability was limited during training, we determined a mini batch size of 16 was suitable for this application. The number of epochs was determined experimentally, aiming to minimize root mean squared error (RMSE) during training while maximizing number of epochs to ensure sufficient adjusting of the CNN’s weights. It is convention to have a dynamic learning rate, so the learn rate schedule was set to piecewise, where the learn rate began at the initial learn rate and monotonically decreased by the learn rate drop factor after each drop period (in epochs; Bengio, 2012). Bengio recommended beginning with a large learning rate and reducing the rate if the training loss does not converge. After testing a few different initial learn rates and drop factors, we found a suitable learn rate schedule to follow. The validation frequency and patience were set to their default values as suggested by MATLAB (The MathWorks, 2020). We used a Dell Precision 5540 (NVIDIA GeForce GTX 1080 GPU, Intel(R) Core(TM) i7-9850H CPU @ 2.60 GHz, 32 GB RAM, Windows 10, 64-bit) to train, validate, and test the CNN. The validation data were shuffled with each epoch to prevent the CNN from over-fitting to the training and validation sets.

**Table 1:** CNN training options

Training option	Setting
Solver	rmsprop
Mini batch size	16
Max epochs	60
Initial learn rate	1e-4
Learn rate schedule	Piecewise
Learn rate drop factor	0.09
Learn rate drop period	10
Validation frequency	50
Validation patience	Inf
Execution environment	gpu
Shuffle	Every epoch

### CNN testing

To evaluate the accuracy of the CNN pipette tip identification used with an iterative proportional feedback controller, we performed a series of experiments over acute brain slices. Specifically, a LabVIEW program randomized the initial pipette location in the field of view (within ±27 μm in the *xy*-plane and ± 6 μm in the *z* direction). The range of training data in the *z* direction was limited to 6 μm since pipette localization error both near the edges of the field of view and out of focus was not observed; thus, did not warrant the excess training data. The CNN used the current image to determine the position of the pipette tip. From that CNN position, the measured error vector was calculated from the origin (center of the field of view, in focus) and used to correct the pipette location back to the origin. The CNN-based pipette tip identification algorithm was run recursively for a predetermined number of iterations (1–4). To determine the true pipette error after iterative correction, the pipette tip was then manually moved to the origin and the change in the manipulator position was saved as the true pipette error.

### Patch clamp experiments

We ran automated patch clamp experiments using a standard electrophysiology rig with four PatchStar micromanipulators on a universal motorized stage (Scientifica, Ltd). We used a peristaltic pump (120S/DV, Watson-Marlow) to perfuse the brain slices with buffer solution. The Multiclamp 700b amplifier (Molecular Devices) and USB-6221 OEM data acquisition board (National Instruments) to collect recordings. We used a pressure control box (Neuromatic Devices) to regulate internal pipette pressure as well as a custom machined chamber with a smaller side chamber for cleaning solution. We followed the cleaning protocol as suggested by Kolb et al. (2016); however, we did not include rinsing in the cleaning protocol because recent literature found that there is no impediment to the whole-cell yield or quality of recording (C. Landry, M. Yip, I. Kolb, WA. Stoy, M.M. Gonzalez, C.R. Forest, unpublished observation). We compared the state-of-the-art cross-correlation method for pipette detection (Kolb et al., 2019) to the CNN method presented here in two different sets of experiments. In order to remove extraneous confounding variables, none of the patch clamp experiments included the cell tracking algorithm used by Kolb et al. (2019), so that any variation because of cell tracking would not affect the success rates of the two pipette identification methods.

### Code accessibility

The code used to train and test the network is included as Extended Data 1, and is also available at https://github.com/mmgxw3/pipetteFindingCNN.

10.1523/ENEURO.0051-21.2021.ed1 Extended Data 1 The MATLAB code. Download Extended Data 1, ZIP file.

### Statistical analysis

To determine statistical significance in success rates, we used the Fischer’s exact test. For the comparison between groups, we used a one-way ANOVA test, and a Tukey’s HSD test. To test for normality, we used a two-sided χ^2^ test that combines skew and kurtosis to test for normality (D’Agostino, 1971; D’Agostino and Pearson, 1973).

## Results

### Validation of pipette position identification

To determine whether the network could accurately identify the pipette tip position over a brain slice, we tested the network on a set of 300 test images, manually annotated with ground truth positions. Representative test images of the pipette over a brain slice, with the CNN position indicated in red, are shown in Figure 1*B*. It is crucial that the CNN errors, $\vec{c}$, are smaller than the true pipette errors, $\vec{t}$, that accumulate during an experiment to ensure that the pipette position error will converge. To demonstrate that the CNN errors, $\vec{c}$, are smaller and more repeatable than the pipette errors from moving to the cleaning bath and back to the sample, $\vec{t}$, these two distributions along each axis are displayed in Figure 1*C*. The mean absolute errors and SDs of the CNN errors for each of the axes are shown in Table 2.

**Table 2:** CNN error from test dataset

Error	$\vec{c}$ (μm)	σ (μm)
*c_X_*	0.39	0.35
*c_Y_*	0.39	0.37
*c_Z_*	0.83	0.88
$\left|\vec{c}\right|$	1.13	0.88

To ensure that the CNN successfully corrected the pipette tip position, we evaluated the network’s ability to converge using the previously described testing workflow. The magnitude of the true pipette error after one to four iterations in the *xy*-plane ($\left|\vec{t_{xy}}\right|$) and *z* direction ($\left|\vec{t_{z}}\right|$) are plotted in Figure 1*D*. While there was a significant difference between the first and second iterations (*p* = 0.001 Tukey’s HSD test), there was no statistical significant difference between the second and third iterations in the *xy*-plane (*p* = 0.49 Tukey’s HSD test). After the second correction, 62% of the attempts were within 1 SD (±0.31 μm) of the target location in the *xy*-plane (*p* = 0.016 D’Agostino test for normality, *α* = 0.05), and 86% of the attempts were within 2 SDs (±0.62 μm), as indicated by the circles in Figure 1*E*. Since the network was able to correct the pipette tip to less than approximately half the diameter of a typical cell (10 μm) in the *xy*-plane with only two iterations of the CNN, we only corrected the pipette position twice for implementation in automated patch clamp experiments. The discretization that is apparent along the *y*-axis is the step size of the micromanipulators, indicating we are approaching the stepper motor encoder resolution. In the *z* direction, 64% of attempts were within 1 SD (0.60 μm) of the target location (*p* = 0.026 D’Agostino test for normality, *α* = 0.05) and 84% of the attempts were within 2 SDs (1.2 μm), which is an acceptable range that we believed would not impair the ability of the pipette to find and patch clamp a cell. The accuracy in the *z* direction was less crucial since the approach method is to descend the pipette from 15 μm above the cell.

### Automated patch clamp experiments

We compared the success rates of the CNN method and the state-of-the-art cross-correlation method on pipette detection, cell detection, and whole-cell recording. Success rates are defined as a fraction of all attempts using the same pipette detection method, independent of whether the previous steps were successful. Success is defined for each of the steps as follows: pipette detection is considered successful when the pipette position can be identified and corrected based on that identification. Cell detection is considered successful when the pipette resistance increases 0.2 MΩ over three consecutive descending 1-μm steps. Whole-cell patch clamp recording is defined by successful cell detection, gigaseal, and break-in. When using the CNN method, two corrections were done after the pipette is brought into the field of view, as previously described. All experiments were done over 5 d, using eight slices from five mice. The numbers of attempts with each method are 32 and 36 for the cross-correlation and CNN methods, respectively. These experiments were done independently, but prepared using the same protocols and solutions to reduce variability in slice health.

**Figure 2.**
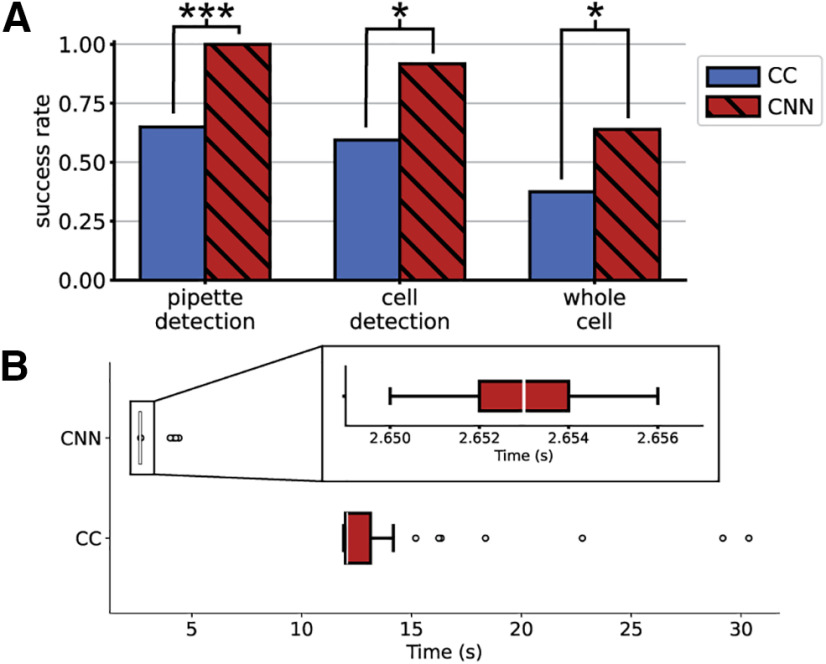
Comparing (***A***) pipette detection, cell detection, and whole-cell success rates and (***B***) time required for cross-correlation and CNN methods (*n* = 32 and ** = 36, respectively). The box width indicates the first and third quartiles, the white line indicates the median, and the whiskers of the box plot indicate the most extreme, non-outlier data points. Using Fischer's exact test; *p ≤ 0.05, ***p ≤ 0.001.

The pipette detection, cell detection, and whole-cell success rates using cross-correlation were 66%, 59%, and 37%, respectively (*n* = 32). The pipette detection, cell detection, and whole-cell success rates using the CNN were 100%, 92%, and 64%, respectively (*n* = 36). These results are summarized in Figure 2*A*. A Fischer’s exact test of the results indicate that the CNN improved the pipette detection success rate by 52% (*p* = 8e-5 Fischer’s exact test), the cell detection success rate by 54% (*p* = 0.001 Fischer’s exact test) and whole-cell success rate by 70% (*p* = 0.05 Fischer’s exact test). Moreover, the CNN method could reliably identify the pipette position regardless of the background noise in the image within 2.71 ± 0.30 s, 81% faster than the average time of the cross-correlation method, as shown in Figure 2*B*.

### Electrophysiology data

**Figure 3.**
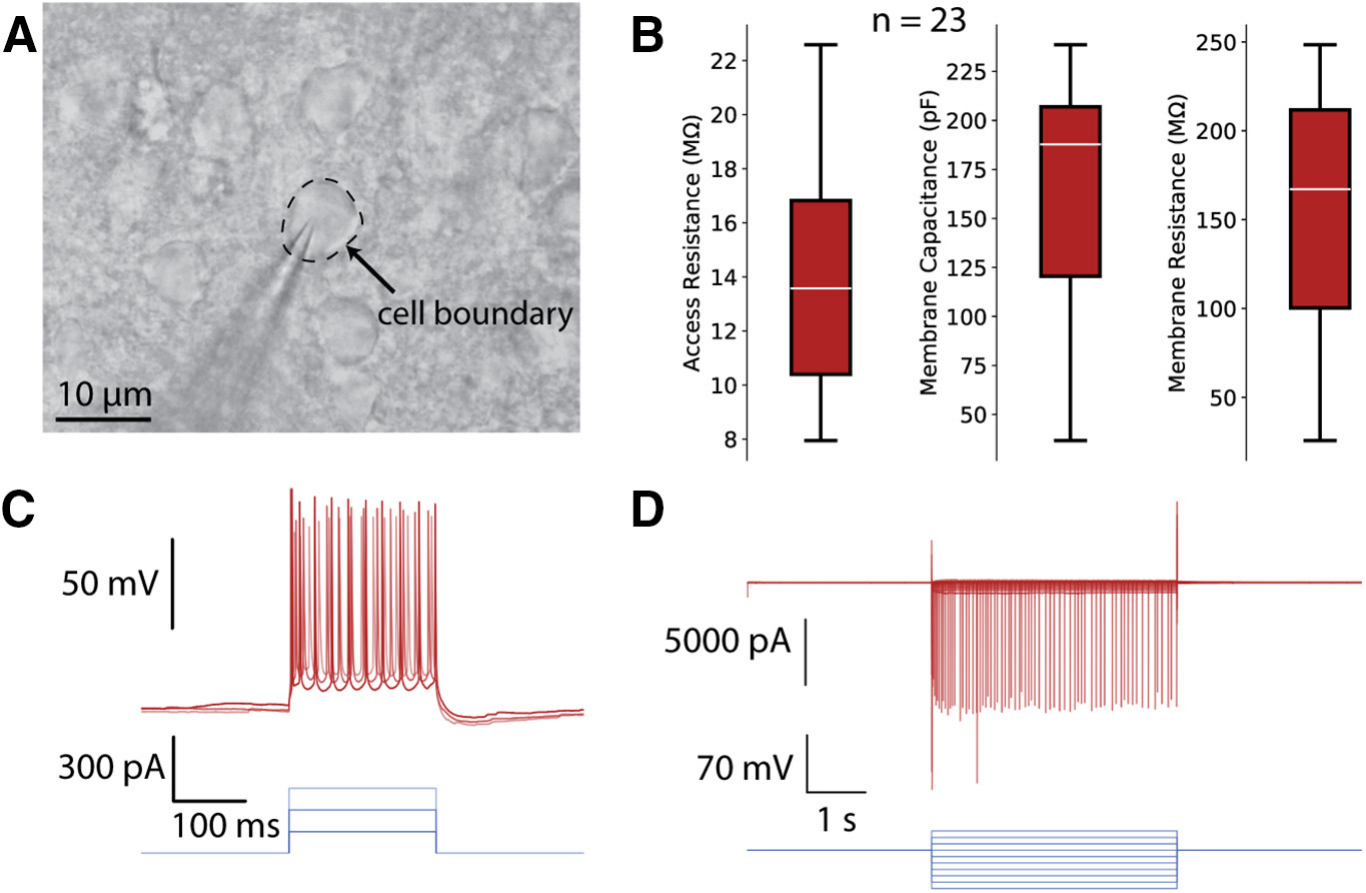
***A***, Example image of a pipette on a neuron during a whole-cell recording. ***B***, Distributions of access resistance, membrane capacitance, and membrane resistance for *n* = 23 successful whole-cell patch clamp recordings using the CNN. The white lines indicate the median, the width of the boxes indicates the first and third quartiles, and the whiskers indicate the range of the data. ***C***, Representative current clamp trace with current injection. ***D***, Representative voltage clamp trace.

The patch clamp experiments done with the CNN method yielded both voltage and current clamp data comparable to the quality of a manual patch clamp expert. An example image of the pipette placed on the cell is shown in Figure 3*A*. The whole-cell recording protocol used was the same as that of Kolb et al. (2019). The distributions of access resistance, membrane capacitance, and membrane resistance are shown in Figure 3*B*. The mean access resistance for recordings performed with the CNN was 14.1 MΩ, well within the accepted range among manual patch clamp experts (<40 MΩ; Kolb et al., 2019). A representative current clamp trace and the corresponding input current injection are displayed in Figure 3*C*. A voltage clamp trace is shown in Figure 3*D*.

## Discussion

One of the primary disadvantages of using the previously reported methods for pipette detection is the lack of reliability. With the cross-correlation method, the pipette tip could be identified for 66% of attempts (*n* = 32), failing because of the difference between the template image and the background noise from the brain slice (Kolb et al., 2019). The deep learning-based pipette detection method presented here offers an accurate and robust method for identifying the pipette tip position in automated patch clamp experiments both over a clear background as well as above a brain slice. Other methods of pipette tip identification have reported accuracy of 12.06 ± 4.3 μm (Long et al., 2015), 3.53 ± 2.47 μm (Koos et al., 2017), and 0.99 ± 0.55 μm (Koos et al., 2021). We reduced the 3D positioning error, using two iterations of our CNN method, to 0.62 ± 0.58 μm. The CNN can more reliably identify the pipette location in the *xy*-plane compared with the *z*-axis. This difference in error distributions is likely because of the fact that small changes of the pipette position in the *z* direction (moving into and out of focus) are less clearly observable when viewing the pipette under the microscope, especially when over a brain slice. However, despite this inherent ambiguity in the pipette tip position, the errors in the *z* direction are still significantly lower than previously reported.

By training a CNN to correct the pipette tip position during automated patch clamp experiments, we improved the success rates of pipette detection to 100% compared with the 66% success rate for cross-correlation. This ability to reliably correct the pipette every time it is in the field of view could be used for automatic calibration or real-time tracking of the pipette’s location for optimization of autopatching protocols. This method also improved the cell detection and whole-cell success rates by 54% and 70%, respectively, compared with the success rates of the cross-correlation method without use of cell tracking, demonstrating the importance of the accuracy and robustness of this crucial step in the autopatching process. Moreover, this CNN method without cell tracking performed similarly with cells 50–60 μm deep (64%) to that of Kolb et al. (2019; 60%), who reported a 60% whole-cell success rate using cross-correlation and cell tracking at the same cell depth (Kolb et al., 2019). Furthermore, the average time required to correct the pipette position using this CNN method is 81% less than the cross-correlation method, averaging 1.6 s per iteration of the CNN identification and movement of manipulators, opening doors to real-time tracking of the pipette tip during automated patch clamp experiments.

There were several limits to this study. For one, we only used one micromanipulator manufacturer (Scientifica). While there may be different error distributions between various manufacturers, we anticipate that this method would still be effective if the modified ResNet101 architecture was trained with new images specific to the objective magnification and manipulator. Further, only pipettes with resistances in the range 3–5 μm were used for training and testing since this range is standard for patch clamp experiments *in vitro*. Pipettes used for other applications, that are significantly narrower or wider, would need more training data to ensure the network could reliably identify the tip’s new geometry. Moreover, use with other objectives would also require collecting new training data. Finally, we omitted the use of cell tracking in the automated patch clamp experiments so that we could isolate errors and measure success rate independently of the cell tracking algorithm.

Future work could use this CNN with cell tracking to simultaneously monitor and correct the pipette location with respect to the cell, potentially leading to even greater whole-cell success rates than previously reported. Moreover, this dual-monitoring could be used to continuously monitor the access resistance and correct the pipette position to maintain this resistance during longer duration experiments. Further, the combined monitoring of the cell and pipette positions may be of great use in multi-electrode automated patch clamp experiments, in which the brain tissue moves more from the simultaneous movement of multiple pipettes in the tissue. This work represents another significant step toward unmanned robotic patch clamp development.

## Synthesis

Reviewing Editor: Alexxai Kravitz, Washington University in St. Louis Decisions are customarily a result of the Reviewing Editor and the peer reviewers coming together and discussing their recommendations until a consensus is reached. When revisions are invited, a fact-based synthesis statement explaining their decision and outlining what is needed to prepare a revision will be listed below. The following reviewer(s) agreed to reveal their identity: Suhasa Kodandaramaiah.

Consensus comments:

1) The paper states that code will be available at a Github link. Code should be live before publication. Training and testing data should also be made available if possible. (Note that eNeuro’s “double blind” review requires them to redact this link during review).

2) Methodological details were insufficient, as detailed in Reviewer comments below. These should be addressed in full.

3) The authors should clarify what is mean in Figure 2 by success rate. ie: what do the percentages for cell detection and whole cell patching mean? For CC method, is the success percentage defined as percentage of all trials (successful and unsuccessful pipette detection) or success percentage within successful pipette detection trials?

4) The patching data shown in Figure 3 shows one representative example. The authors should provide group metrics for successfully patched cells so a reader can confirm the quality and variance of the data collected with this approach.

5) More information on the size of field of view and the angle of approach should be provided, as well as information on how changes in these variables affect the accuracy of the pipette detection. If a new pipette, or a new angle of approach is used, does the algorithm require re-training?

6) While a comparison is made with the cross correlation method, there is no citation for which cross correlation method was used.

Original reviewer comments:

*****************

Reviewer 1

*****************

There has been progress made in recent years automating patch clamping in vivo and in vitro, and increasing sophisticated algorithms that are being used for guide patch pipettes to target cells of interest. These advances could in the very near future lead to truly human out of the loop autonomous patch clamping robots. One sub-issue with these automation efforts specific to image guided patching using DIC optics in vivo is need to accurately determine the location of the patch pipette tip using computer vision. This paper deals with this issue. While this is a niche application, patch clamp electrophysiology is still the gold standard, and a faster, more accurate pipette tip detect algorithm that can be incorporated into fully autonomous patch clamp robots could be beneficial to the field. Particularly if this can be combined wire recent advances in deep learning based cell detection algorithms being developed for automated patch clamping systems. Overall, there are however key methodological details that are missing (See specific comments below). The paper relies on testing pipettes oriented at a single angle, and it is not clear currently, how much variability was incorporated in both the training dataset and the final experiments. As a final comment, the paper is currently written for an audience that is already well versed in automated patch clamping, with a very narrow focus pipette tip detection - which makes it unsuitable in its current form for a broader neuroscience audience.

Major Comments

"Light scattering from the brain tissue induces significant noise in the image and renders these methods practically useless since they rely on a clear image of the pipette” Strong statement considering these approaches are still useful for patching in cultured cell preparations.

- A major claim of the paper is that previous pipette tip localization work is prone to errors arising due to variations in lighting. While they report top-line numbers, there is little detail with regards to the testing conditions with the two methods. What were the lighting an imaging conditions used in training dataset and the experiments? In other works, will the algorithm work similar to the results shown in this work across multiple groups? There is little methodological details provided.

- Along the same lines, it is unclear how the training dataset was acquired, annotated.

- For this to be easily implemented in other groups, both training datasets and experiment datasets should be made available (I know these may be TBs of data).

- There are methodological details missing in the description of the data pre-processing step.

- The health of the slice can have a significant effect on eventual success rate of whole cell patch clamping. In figure 2, a comparison of CNN and cross correlation methods are provided. It is not clear how the trials were conducted. Were both methods attempted on multiple cells in the same slice? Or were these independent experiments? How many replicates does the data shown represent?

- Success rates for pipette detection, cell detection and whole cell patching are provided. Unclear what percentages for cell detection and whole cell patching mean here. i.e., for CC method, is the success percentage defined as percentage of all trials (successful and unsuccessful pipette detection) or success percentage within successful pipette detection trials. If it is the latter, this is a surprising result as you wouldn’t expect such a large increase in success rate of patching. Do the authors have an explanation for this? If it is the latter, the increase in success rate is entirely due to the better pipette positioning.

- The number of experimental sessions, replicates information is missing in the discussion or success rates.

- As described, the convolution neural network algorithm is only viable within +/-27μm of the origin in the xy plane and +/-6μm of “optimal focus” in the z direction. How late is the field over view relative to this area? I’m not sure if this is a small region around the center of the image or significant portion of the image. How is optimum focus defined as? Depending on the numerical aperture of the lens, that itself may be a few micrometers in range. What happens is the pipette is not at optimal focus?

- The patching data shown in Figure 3 is entirely representative.

- While a comparison is made with the cross correlation method, there is no citation for which cross correlation method was used. There is one instance where they discuss multiple previous methods.

- Was the algorithm tested for pipettes are different approach angles (which is common is slice patching experiments)?

- How long does the acquisition of training dataset and training take? The 81% reduction is time taken to detect the pipette tip is significant and impressive.

Minor Comments

- Use of informal ‘micron’ instead of micrometer.

- Ref. 20 Koos et al is now a published article. I believe Koos Nat Comm 2021.

- In order to remove extraneous confounding variables, 153 none of the patch clamp experiments included the cell tracking algorithm used by Kolb et al. so that any variation due to cell tracking would not affect the success rates of the two pipette identification methods There are three papers authored by Kolb here. A more specific citation is needed.

- With the cross correlation method, the pipette tip could be identified for 66% of attempts 216 (n = 32), failing due to the difference between the template image and the background noise from the brain slice. Please insert citation (I know it is earlier).

- Line 117: RAM itself is a Random-Access Memory no need to say RAM memory

- p vector is cited in the text, but figure 1 doesn’t have a p vector

- Sentence in abstract should read “whole-cell [patch clamp] success rate

- Y-axis label is missing in Figure 2b

*****************

Reviewer 2

*****************

Machine learning-based pipette positional correction for automatic patch clamp in vitro

The paper is describing a new method for the identification of the pipette tip during patch-clamp experiments. The method uses a convolutional neural network (CNN) based on ResNet101 to identify the pipette tip online during a patch attempt. The reported accuracy is 0.62 μm. There are also reported improvements in the whole-cell patch-clamp success rate.

Overall, it is an important technological development. Pipette tip detection is crucial for automated patch clamp. Using a CNN network for recognition and detection of the pipette tip is definitely important, considering the authors provide a detailed description of the process and make the code available.

However, there are several issues related to this paper:

1) Not sure if this algorithm can detect a pipette tip “de novo.” One would expect the same pipette tip detection algorithm to be used for both initial tip detection and then for correction.

2) The second related issue comes from the fact that previous pipette tip detection methods have been published, which reduces the novelty of this paper.

3) Finally, this paper describes only the positional correction of the detected pipette tip and doesn’t really have any other additional data or automated patch clamp development, including any novel neuroscience data.
